# Pharmacoeconomic evaluation of first-line tislelizumab for extensive-stage small cell lung cancer using a comparative validation of traditional survival and machine learning models

**DOI:** 10.3389/fpubh.2026.1841510

**Published:** 2026-06-17

**Authors:** Yang Li, Yang Zou, Canhua Liang, Ziwei Feng, Shaohuan Lu, GuangZhao Wang, Guangyi Meng

**Affiliations:** Department of pharmacy, The First People's Hospital of Yulin, Guangxi, Yulin, China

**Keywords:** cost-effectiveness analysis, DeepSurv, extensive-stage small cell lung cancer, machine learning, random survival forest, tislelizumab

## Abstract

**Objective:**

To evaluate the cost-effectiveness of first-line tislelizumab plus chemotherapy for extensive-stage small cell lung cancer (ES-SCLC) within the Chinese healthcare context. Furthermore, this study aims to comparatively validate the impact of traditional survival models vs. advanced machine learning models on the robustness of research findings, thereby providing refined empirical evidence for healthcare decision-making.

**Methods:**

Based on data from the Phase III RATIONALE-312 clinical trial, a partitioned survival model (PSM) was constructed with a 10-year time horizon. From the perspective of the Chinese healthcare system, only direct medical costs were included. A willingness-to-pay (WTP) threshold was set at three times the 2025 per capita GDP of China (298,995 CNY/QALY). A dual-validation strategy was employed for survival extrapolation: a base-case analysis using the optimal parametric model (Log-logistic distribution) selected via Akaike Information Criterion/Bayesian Information Criterion (AIC/BIC) criteria, followed by a comparative validation using DeepSurv deep learning and random survival forest (RSF) models utilizing a simplified feature set. Uncertainty was assessed through one-way sensitivity analysis and probabilistic sensitivity analysis (PSA) using 1,000 Monte Carlo simulations.

**Results:**

The base-case analysis (Log-logistic model) revealed that, compared with chemotherapy alone, the tislelizumab group yielded an incremental cost of 101,506.9 CNY and incremental quality-adjusted life years (QALYs) of 0.4044, resulting in an incremental cost-effectiveness ratio (ICER) of 251,030.5 CNY/QALY, which remains below the WTP threshold. Machine learning validation demonstrated exceptional consistency, with ICERs of 261,718.45 CNY/QALY for the DeepSurv model and 248,299.41 CNY/QALY for the RSF model. Sensitivity analysis identified the utility of progression-free survival (PFS) and the unit price of tislelizumab as the primary drivers of the model. PSA confirmed that the probability of tislelizumab being cost-effective exceeded 85% across all three survival-fitting logics, reaching a maximum of 94.90%.

**Conclusion:**

First-line tislelizumab plus chemotherapy demonstrates a significant cost-effectiveness advantage for ES-SCLC in China. The integration of machine learning survival models effectively reduced the inherent uncertainties of non-linear extrapolation in immunotherapy data, confirming the robustness of the conclusions. These findings provide a solid empirical basis for national health insurance negotiations and precision clinical applications.

## 1 Introduction

Small-cell lung cancer (SCLC) is a highly aggressive neuroendocrine malignancy, accounting for approximately 15% of all lung cancer cases (1). Among these, extensive-stage SCLC (ES-SCLC) represents a high proportion of initial diagnoses and is characterized by rapid proliferation, early metastatic potential, and a dismal prognosis, with 2-year survival rates typically falling below 15% (2). For decades, etoposide combined with platinum has remained the standard first-line therapy for ES-SCLC; however, median survival has historically struggled to exceed 10 months (3). In recent years, the integration of immune checkpoint inhibitors (ICIs) with chemotherapy has led to transformative breakthroughs in the first-line setting for ES-SCLC. The RATIONALE-312 study, a large-scale Phase III clinical trial conducted in the Chinese population, demonstrated that the addition of tislelizumab to conventional chemotherapy significantly extends both overall survival (OS) and progression-free survival (PFS) in patients with ES-SCLC, offering a novel therapeutic option for patients in China (4). Nevertheless, the high acquisition costs of immunotherapeutic agents and the resulting economic burden of long-term treatment present significant challenges for healthcare resource allocation in clinical practice.

In pharmacoeconomic evaluation research, the fundamental challenge in constructing partitioned survival models (PSM) lies in the accurate extrapolation of survival data from the “observation period” to the “full life cycle”(5). Traditional parametric survival models, such as the Weibull, Log-logistic, and Gompertz distributions, are essentially parameter estimates based on *a priori* distribution assumptions. Their validity is highly dependent on the monotonicity of the hazard function or specific morphological assumptions (6). However, clinical data involving ICIs frequently exhibit non-proportional hazard characteristics or a “long-tail effect” in survival curves. Traditional models often struggle to capture these complex non-linear evolutionary trends and are susceptible to individual patient heterogeneity. This can lead to significant biases in extrapolation results and an inability to capture the intricate non-linear relationships within clinical data (7, 8). With the deep integration of computational medicine and data science, machine learning algorithms—represented by random survival forest (RSF) and the DeepSurv deep learning model—have demonstrated substantial technical advantages (9). RSF, a non-parametric ensemble learning method, constructs multiple decision trees and utilizes random feature subsets for node splitting to adaptively learn interactions within survival data without pre-specifying distribution types or hazard ratios. This approach offers good resistance to overfitting and predictive robustness (10). Meanwhile, the DeepSurv model integrates deep neural networks with Cox proportional hazards logic, utilizing a multi-layer perceptron architecture to automatically extract high-dimensional non-linear representations of patient baseline characteristics. This allows for more flexible fitting of complex clinical survival trajectories, surpassing the limitations of traditional regression models that require manual definition of interaction terms (11).

Utilizing data from the RATIONALE-312 trial, this study conducts an economic analysis from the perspective of the Chinese healthcare system, employing standard parametric distributions while incorporating advanced machine learning survival models for comparative validation. We aim to evaluate the cost-effectiveness and robustness of the tislelizumab regimen across different modeling frameworks, thereby identifying and mitigating the risks of uncertainty associated with model selection. This research provides a robust empirical foundation for national health insurance negotiations and clinical decision-making in China.

## 2 Methods

### 2.1 Target population and treatment regimens

The patient characteristics and therapeutic interventions in this study were derived from the RATIONALE-312 trial, a Phase III, multicenter, randomized, double-blind, placebo-controlled clinical trial conducted in China. Eligible patients were ≥18 years of age with histologically or cytologically confirmed ES-SCLC according to the American Joint Committee on Cancer 7th edition diagnostic criteria. Patients were required to have an Eastern Cooperative Oncology Group performance status score of 0 or 1, an expected survival of ≥12 weeks, and no prior systemic therapy for ES-SCLC. A total of 457 patients were enrolled and randomized 1:1 in a double-blind manner to receive either tislelizumab or placebo. Both cohorts received four cycles of induction chemotherapy comprising etoposide (100 mg/m^2^ intravenously on days 1–3 of each 21-day cycle) and platinum 75 mg/m^2^ or carboplatin at an AUC of 5), with 200 mg of tislelizumab or placebo administered on day 1 of each cycle. Following induction, the tislelizumab group continued maintenance therapy while the placebo group received a placebo until disease progression, loss of clinical benefit, unacceptable toxicity, or withdrawal of consent, with a cycle duration of 3 weeks. Radiographic assessments were performed every two cycles.

### 2.2 Model Structure

**Figure 1:**
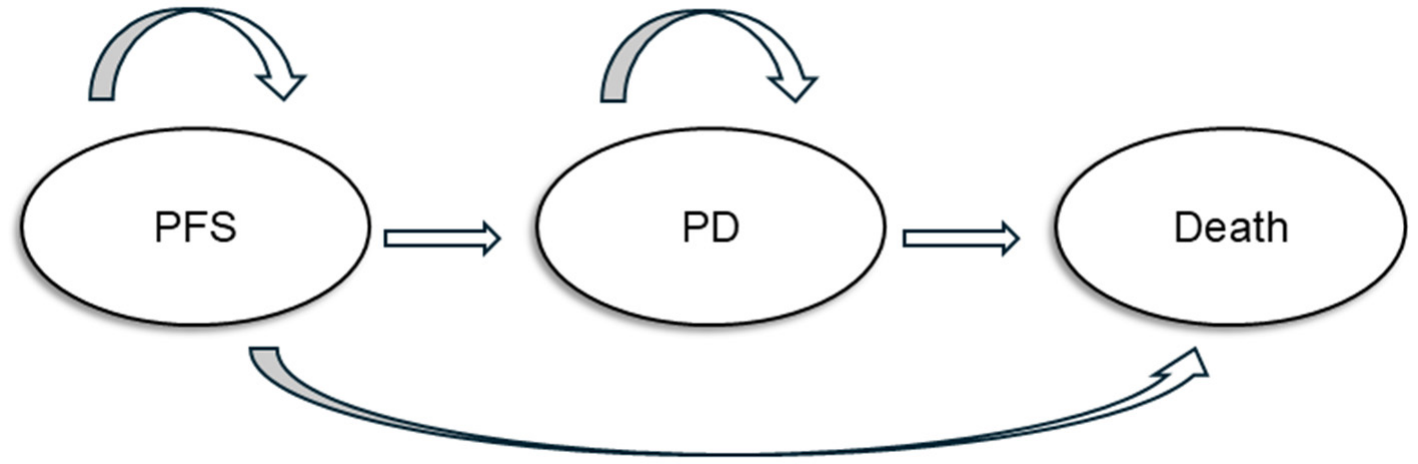
Structure of partitioned survival model.

A PSM was constructed using TreeAge Pro software (2022 version). The model defined three mutually exclusive health states: PFS, PD, and death (Figure 1). At the beginning of the simulation, all patients were assumed to be in the PFS state. The model utilized a cycle length of 3 weeks and a total time horizon of 10 years (12). Costs and health utilities were discounted at an annual rate of 5% to calculate the total costs and quality-adjusted life years (QALYs), which were subsequently used to derive the incremental cost-effectiveness ratio (ICER). The willingness-to-pay (WTP) threshold was established at three times the 2025 per capita gross domestic product (GDP) of China, equivalent to 298,995CNY/QALY. The economic value of the tislelizumab regimen relative to chemotherapy alone was evaluated by comparing the resulting ICER against this threshold (13).

### 2.3 Survival analysis

**Figure 2:**
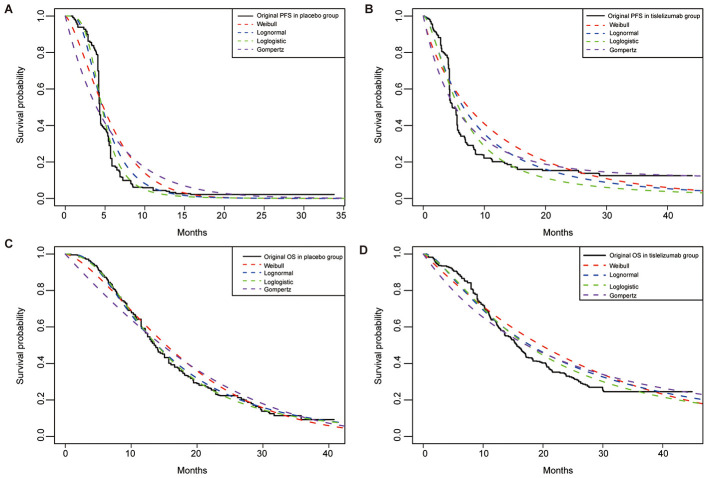
Fitting extrapolated K-M curves using parametric models **(A)**. PFS curve of the placebo group, **(B)**. PFS curve of the tislelizumab group, **(C)**. OS curve of the placebo group, **(D)**. OS curve of the tislelizumab group.

**Figure 3:**
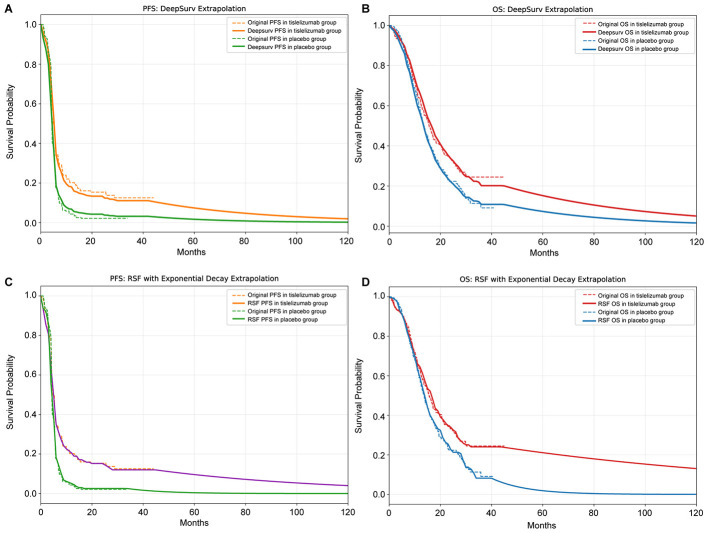
Fitted and extrapolated survival curves using DeepSurv and RSF models. **(A)**. Fitted PFS curve using DeepSurv model, **(B)**. Fitted OS curve using DeepSurv mode, **(C)**. Fitted PFS curve using RSF model, **(D)**. Fitted OS curve using RSF model.

First, survival data for PFS and OS were extracted from the RATIONALE-312 trial using GetData Graph Digitizer software. Subsequently, individual patient data (IPD) were reconstructed using the survHE package in R (version 4.4.0). Survival curves were fitted using Gompertz, Log-logistic, Log-normal, and Weibull standard parametric models and extrapolated beyond the clinical trial observation period (Figure 2) (14). To evaluate the impact of traditional parametric vs. machine learning models on economic outcomes, two mainstream machine learning survival models—RSF and DeepSurv—were incorporated for comparative analysis. It should be noted that, DeepSurv and RSF were employed in this study as highly flexible non-parametric function approximators for survival curve fitting, rather than multivariable predictive models for individual risk stratification. Both machine learning models were fitted in a Python environment (version 3.10.19) based on the reconstructed IPD. The RSF model was implemented via the scikit-survival package; as an ensemble learning method, it bypasses the need for predefined survival distributions and predicts time-dependent survival probabilities through an ensemble of decision trees. This approach demonstrates robust anti-overfitting capabilities and effectively manages non-linear relationships and interactions within survival data. The DeepSurv model was implemented using the torch, pycox, and torchtuples packages. Based on a deep neural network architecture, DeepSurv captures high-dimensional non-linear associations via multi-layer perceptrons without relying on any distribution assumptions, thereby offering greater flexibility in fitting complex trajectories and accommodating highly heterogeneous clinical data (Figure 3). The hyperparameter configurations used in our study are summarized in Supplementary Table S1. Survival probabilities from all models were utilized to construct the PSM and calculate corresponding economic indicators for comparative analysis. To objectively assess the reliability of survival extrapolations, a dual-validation strategy was employed. For traditional parametric models, the optimal distribution was selected based on the Akaike Information Criterion (AIC) and Bayesian Information Criterion (BIC) (12). Meanwhile, the predictive performance of the machine learning models was quantified using the concordance index (C-index) and Integrated Brier Score (IBS) (15). To address the risk of potential overfitting, a non-parametric bootstrapping approach with 1,000 resamples was implemented for internal validation.

### 2.4 Costs and health utilities

From the perspective of the Chinese healthcare system, only direct medical costs were incorporated, including expenditures for drugs, drug administration, tumor imaging, follow-up, and subsequent supportive care. Drug acquisition costs were sourced from the centralized procurement prices publicized by the local drug and medical consumable bidding and procurement management systems (https://ybwt.ybj.gxzf.gov.cn/), updated through February 28, 2026. To estimate the requisite therapeutic dosages, the following baseline patient characteristics were assumed: a body weight of 65 kg, a body surface area of 1.72 m^2^, and a creatinine clearance rate of 70 ml/min (16). To streamline model calculations, the costs of adverse events (AEs) were applied only during the first cycle of either the PFS or PD states. Inclusion was restricted to severe AEs (Grade≥3) with an incidence rate ≥10%, with frequency data derived from the RATIONALE-312 trial (4). All other cost parameters were obtained from published literature. Key model inputs are detailed in Table 1.

**Table 1:** Base-case key model inputs.

Parameter	Baseline value	Lower limit	Upper limit	Distribution	Reference
Drug Cost (CNY/cycle)					
Tislelizumab	2,755	2,204	3,306	Gamma	National Databases
Cisplatin	82.21	78.82	149.02	Gamma	National Databases
Carboplatin	160.62	125.21	750.5	Gamma	National Databases
Etoposide	125.11	92.88	157.34	Gamma	National Databases
Topotecan	551.64	551.58	4310.75	Gamma	National Databases
Cost of terminal care per patient	17 727.50	14 182.00	21 273.00	Gamma	(17)
Cost of administration per cycle	256.32	205.06	307.58	Gamma	(18)
Cost of laboratory per cycle	466	372.8	559.2	Gamma	(19)and average prices of each health service from health service projects in 29 Chinese provinces
Cost of tumor imaging per cycle	3609.84	2887.87	4331.81	Gamma	(18)
Cost of best supportive treatment per cycle	3 155.50	2 524.40	3 786.59	Gamma	(20)
Cost of AEs (CNY/cycle)					
Anemia	3 536.60	2 829.28	4 243.92	Gamma	(21)
Thrombocytopenia	10 555.00	8 444.00	12 666.00	Gamma	
Leukopenia	3 099.60	2 479.68	3 719.52	Gamma	
Neutropenia	3 069.67	2 455.74	3 683.60	Gamma	
Risk of AEs in tislelizumab group					
Anemia	0.16	0.13	0.19	Beta	(4)
Thrombocytopenia	0.19	0.15	0.23	Beta	
Leukopenia	0.24	0.19	0.29	Beta	
Neutropenia	0.14	0.11	0.17	Beta	
Risk of AEs in placebo group					
Anemia	0.17	0.14	0.2	Beta	(4)
Thrombocytopenia	0.25	0.2	0.3	Beta	
Leukopenia	0.27	0.22	0.32	Beta	
Neutropenia	0.21	0.17	0.25	Beta	
Utility PFS	0.67	0.54	0.81	Beta	(22)
Utility PD	0.47	0.38	0.57	Beta	
Utility anemia	0.07	0.06	0.09	Beta	(23, 24)
Utility thrombocytopenia	0.02	0.02	0.03	Beta	
Utility leukopenia	0.11	0.09	0.14	Beta	
Utility neutropenia	0.2	0.16	0.24	Beta	
Proportion of subsequent anticancer therapy in placebo group	0.67	0.54	0.8	Beta	(4)
Proportion of subsequent anticancer therapy in tislelizumab group	0.55	0.44	0.66	Beta	
Weight (Kg)	65	52	78	Normal	(4)
Body surface area (m^2^)	1.72	1.38	2.06	Normal	
Area under the curve (mg/ml/min)	5	4	6	Uniform	
Serum creatinine(mg/dl)	1	0.8	1.2	Uniform	
Discount rate	0.05	0	0.08	Beta	(25)

### 2.5 Sensitivity analysis

To evaluate the robustness of the findings against parameter variations, both one-way sensitivity analysis (OWSA) and probabilistic sensitivity analysis (PSA) were performed. Key parameters included drug costs, health utilities, and the risk of AEs. The discount rate was varied between 0 and 8%. While the ranges for utility values in the PFS and PD states were derived from published literature, all other parameters were adjusted within ±20% of their base-case values. The results of the OWSA are presented as tornado diagrams. For the PSA, second-order Monte Carlo simulations were conducted with 1,000 iterations. Parameters were randomly sampled from specified probability distributions to illustrate the potential impact of multi-parameter uncertainty on the analysis results via cost-effectiveness acceptability curves and cost-effectiveness scatter plots. Furthermore, in order to assess the impact of long-term extrapolation predictions on the stability of the model, we conducted a scenario analysis by reducing the time range from 10 years to 5 years.

## 3 Results

### 3.1 Model evaluation and verification

Prior to the cost-effectiveness analysis, a multidimensional performance evaluation of the survival fitting models was conducted. Among the parametric survival models, the Log-logistic distribution was identified as the optimal fitting distribution based on AIC and BIC criteria (Table 2 and Supplementary Table S2). In the evaluation of machine learning models, both DeepSurv and RSF exhibited a C-index of approximately 0.52 for OS and PFS predictions. Although this indicates limited discriminative performance at the individual level, this is primarily attributable to the use of reconstructed IPD from the RATIONALE-312 clinical trial, which lacks rich baseline covariates beyond treatment assignment, thereby constraining the models' ability to rank individual risks. However, in the present study, the primary purpose of the machine learning models was to perform long-term extrapolation of population-level survival curves. Therefore, the IBS, which reflects the accuracy of survival probability fitting, is considered a more informative and relevant evaluation metric. In the present study, the IBS for the DeepSurv model in PFS prediction was only 0.0895, suggesting that the models demonstrated good reliability in survival curve fitting and probability calibration, and were able to adequately capture the dynamic evolution of survival data. Detailed evaluation metrics are provided in Table S3.

**Table 2:** Survival curve optimal fitting and parameter distribution.

Group	Best fitted distribution	AIC	BIC	shape-γ	scale-α
PFS of placebo group	Loglogistic	1995.034	2003.279	3.8442	4.6129
PFS of tislelizumab group	Loglogistic	2578.537	2586.787	1.6692	5.7917
OS of placebo group	Loglogistic	3115.87	3124.115	2.2952	13.9101
OS of tislelizumab group	Loglogistic	2929.523	2937.772	1.5439	17.3555

### 3.2 Base-case analysis results

In this study, cost-effectiveness analyses for the tislelizumab plus chemotherapy group vs. the placebo plus chemotherapy group were conducted based on the optimal parametric model (Log-logistic) and two machine learning validation models (DeepSurv and RSF), with results summarized in Table 3 and Supplementary Table S4. Under the Log-logistic model (base-case analysis), the total costs were 254,736.1 CNY for the tislelizumab group and 153,229.2 CNY for the placebo group, yielding an incremental cost of 101,506.9 CNY. The tislelizumab group achieved 1.1117 QALYs, compared with 0.7073 QALYs in the control group, representing an incremental gain of 0.4044 QALYs. The ICER was calculated as 251,030.5 CNY/QALY, which remains below the WTP threshold of 298,995 CNY/QALY.

**Table 3:** The results of cost-effectiveness.

Model	Group	Total cost (CNY)	Incremental cost (CNY)	QALYs	Incremental QALY	ICER/(CNY/QALY)
Log-logistic	Placebo group	15,3229.2	101,506.90	0.7073	0.4044	251,030.50
	Tislelizumab group	25,4736.1		1.1117		
DeepSurv	Placebo group	13,0453.03	69,000.06	0.5298	0.2636	26,1718.45
	Tislelizumab group	199453.09		0.7934		
RSF	Placebo group	114,264.31	115,092.34	0.4566	0.4635	248,299.41
	Tislelizumab group	22,9356.65		0.9201		

Validation using machine learning models revealed ICERs of 261,718.45 CNY/QALY for the DeepSurv model and 248,299.41 CNY/QALY for the RSF model, both of which were below the WTP threshold. Across the three survival-fitting frameworks, the ICERs for tislelizumab plus chemotherapy vs. chemotherapy alone demonstrated high consistency and were robustly lower than the WTP threshold. These findings indicate that the tislelizumab-based regimen possesses a definitive cost-effectiveness advantage in the first-line treatment of ES-SCLC.

### 3.3 One-way sensitivity analysis

**Figure 4:**
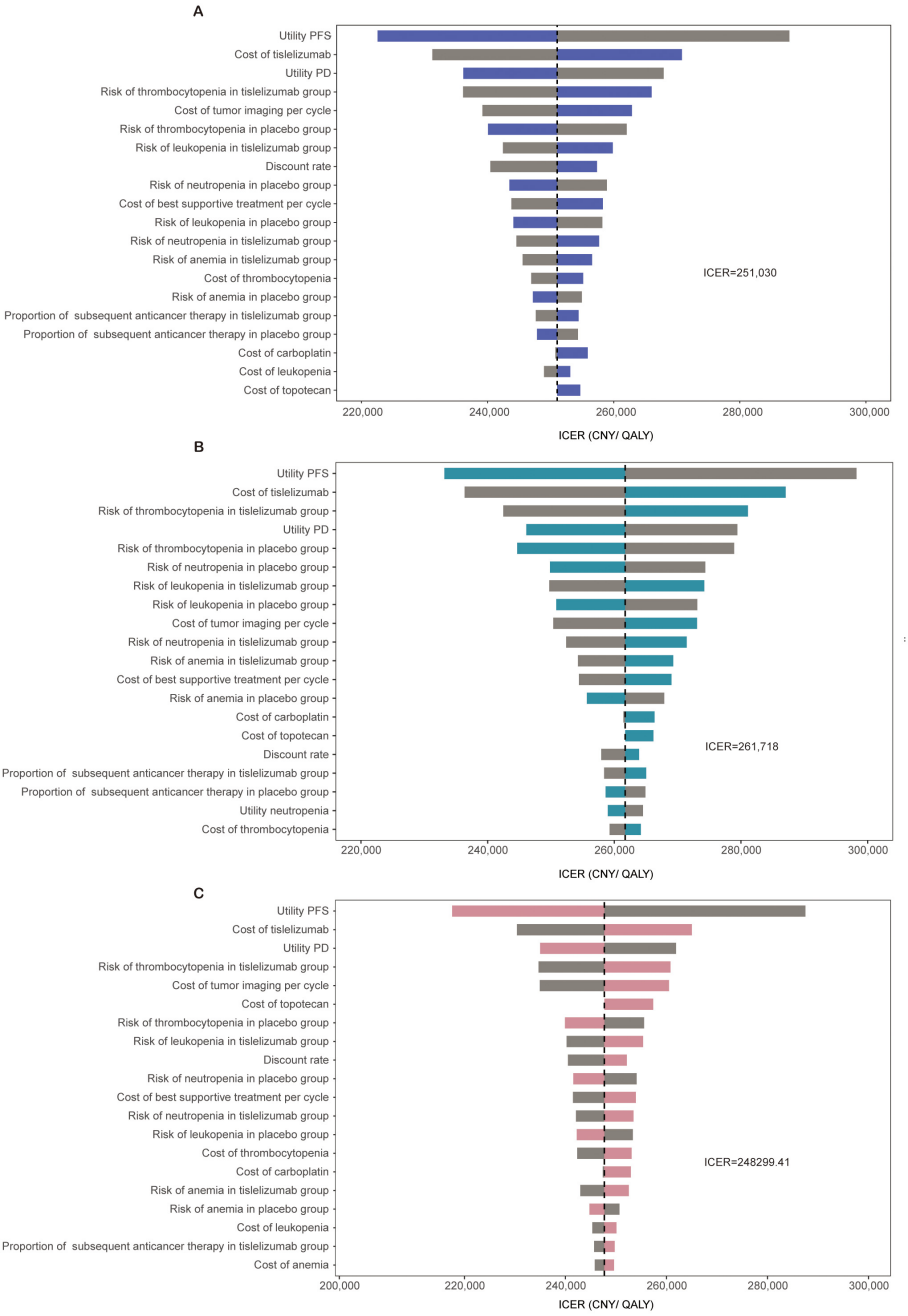
Tornado plots of one-way sensitivity analysis for the three models. **(A)**. One-way sensitivity analysis for the Log-logistic model, **(B)**. One-way sensitivity analysis for the DeepSurv model, **(C)**. One-way sensitivity analysis for the RSF model.

The impact of individual parameters on the ICER was assessed through OWSA, with the results illustrated in Figure 4 and Supplementary Table S5. Across all three models, the analysis consistently identified the utility value of PFS, the acquisition cost of tislelizumab, the utility value of PD, and the incidence of thrombocytopenia during treatment as the primary drivers of the ICER. Nevertheless, within the plausible ranges of all parameters, the ICER remained robustly below the WTP threshold of 298,995 CNY/QALY. Conversely, the disutilities associated with AEs, such as anemia and thrombocytopenia, exerted a negligible influence on the overall ICER.

### 3.4 Probabilistic sensitivity analysis

**Figure 5:**
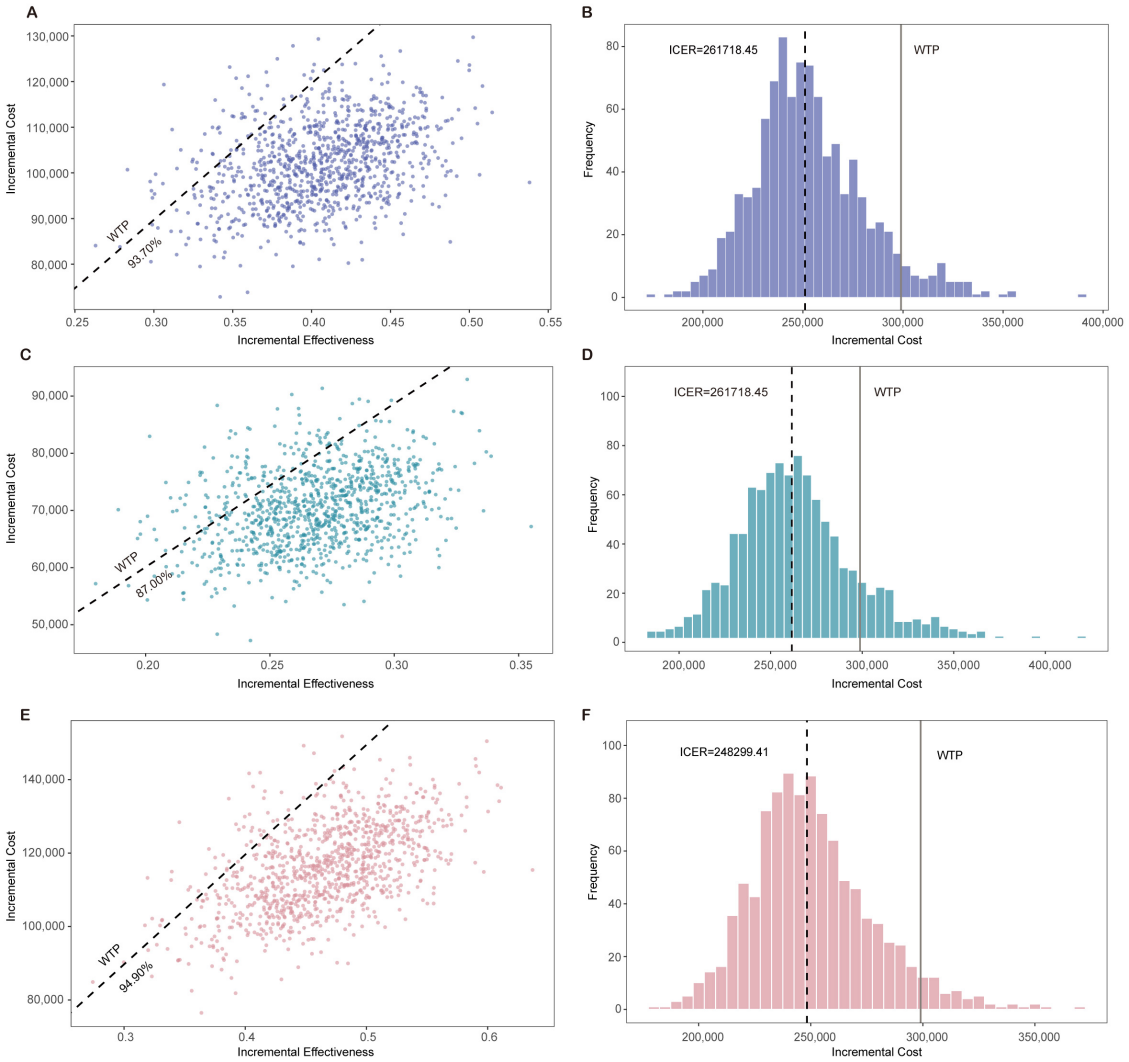
Probabilistic sensitivity analysis results for the three models. **(A)**. Scatter plot for the Log-logistic model, **(B)**. Histogram for the Log-logistic model, **(C)**. Scatter plot for the DeepSurv model, **(D)**. Histogram for the DeepSurv model, **(E)**. Scatter plot for the RSF model, **(F)**. Histogram for the RSF model.

PSA was conducted using 1,000 Monte Carlo simulations, with 1,000 iterations successfully completed (100% effective rate). Detailed results are presented in Figure 5 and Supplementary Figure S1. The cost-effectiveness scatter plots revealed that 93.7% of the simulation iterations for the Log-logistic model were distributed below the WTP threshold line. For the DeepSurv and RSF models, 87.00% and 94.90% of the iterations, respectively, were located below the WTP threshold line. ICER distribution histograms further substantiated the robustness of the base-case results, with ICER values predominantly clustered near the base-case estimates and consistently remaining below the WTP threshold. Across the three survival-fitting frameworks, PSA results consistently indicated a high probability of cost-effectiveness for tislelizumab plus chemotherapy compared with placebo plus chemotherapy for the first-line treatment of ES-SCLC. This confirms the robust nature of the research conclusions under conditions of parameter uncertainty.

## 4 Discussion

Based on data from the RATIONALE-312 trial, this study utilized a PSM to evaluate the cost-effectiveness of first-line tislelizumab plus chemotherapy vs. chemotherapy alone for ES-SCLC. A pivotal innovation of this research lies in the comparative assessment of how traditional parametric survival models and machine learning models influence economic evaluation outcomes. During model selection, although the Log-logistic distribution performed optimally according to statistical metrics (AIC/BIC), such standard parametric models are fundamentally “strong-assumption” models, whose extrapolation efficacy is heavily contingent upon the predefined shape of the hazard function. To further verify the robustness of our conclusions, this study introduced the DeepSurv deep learning model and the RSF model as validation anchors. DeepSurv captures high-dimensional non-linear associations within survival data via multi-layer perceptrons, whereas RSF leverages ensemble learning algorithms to enhance predictive anti-overfitting capabilities without requiring predefined distributions. Our findings indicate that despite the disparate underlying mathematical logics of these three frameworks (standard parametric distribution vs. neural network vs. forest ensemble), the calculated ICER values (248,000–262,000 CNY/QALY) consistently fell within the WTP threshold with minimal numerical variance. This convergence of results strongly demonstrates that the economic value of tislelizumab is not an artifact of an accidental fit to a specific mathematical distribution but is instead underpinned by clinically meaningful benefits. Moreover, it validates the feasibility of employing machine learning models as validation tools for survival extrapolation. OWSA further revealed that the utility values for the PFS state, the acquisition cost of tislelizumab, and the utility values for the PD state are the primary drivers of the ICER. Within a plausible range of parameter fluctuations (±20%), the ICER remained below the WTP threshold, indicating that tislelizumab maintains its cost-effectiveness advantage across various parameter assumptions. In clinical practice, further optimization of drug pricing, mitigation of adverse reactions, and enhancement of patient quality of life will be critical to the cost-effectiveness performance of this therapeutic regimen.

The findings of this study exhibit high consistency with the results reported by Fan et al., which were also based on the RATIONALE-312 trial, further confirming the cost-effectiveness of this regimen from the perspective of the Chinese healthcare system (26). Although the ICER reported by Fan et al. under the PSM model (206,915.66 CNY/QALY) was slightly lower than that in our study, this marginal discrepancy primarily stems from dynamic updates in drug pricing and differences in model selection. Specifically, our study utilized the latest procurement prices updated through February 2026, which more accurately reflect current pharmaceutical market conditions and real-world costs following national insurance negotiations. Furthermore, the integration of machine learning algorithms, such as DeepSurv and RSF, enabled the capture of more complex non-linear evolutions in survival data, thereby enhancing the robustness of the extrapolation results.

In the current landscape of ES-SCLC treatment, tislelizumab faces intense competition from other ICIs. Regarding horizontal comparisons, with the intensive development of domestically developed ICIs in the ES-SCLC field, tislelizumab encounters direct competition from regimens such as toripalimab and socazolimab. Evaluations based on the EXTENTORCH trial indicate that the ICER for toripalimab ranges from 142,000 to 210,000 CNY/QALY. Moreover, the selection of specific beneficiary populations via biomarkers, such as the A11+/B62- genotype, could further enhance its cost-effectiveness (27, 28). This suggests that future research should further explore the cost-effectiveness of tislelizumab across different biomarker stratifications to achieve more efficient allocation of healthcare resources. Concurrently, research by Tang et al. on socazolimab also confirms the cost-effectiveness advantages of domestic PD-L1 inhibitors in this therapeutic area (29). A network meta-analysis of eight Phase III clinical trials of ICIs for ES-SCLC conducted by Wen et al. (30) revealed that while certain combination regimens (e.g., benmelstobart plus anlotinib and chemotherapy) ranked highest in terms of efficacy for extending OS and PFS, their prohibitive combination costs often limited their economic performance. In contrast, the tislelizumab regimen achieves an optimal balance among clinical benefit, safety, and economic burden. According to the cost-effectiveness results from this network meta-analysis, the tislelizumab regimen attained the second-highest QALYs (1.27 years) and the second-lowest treatment costs ($52,273.46), rendering its ICER highly attractive among comparable agents

Although this study enhances the reliability of its conclusions through multi-model cross-validation, several limitations remain. First, the utility values for each health state were sourced from published domestic and international literature rather than direct EQ-5D measurements from the RATIONALE-312 trial. This may not fully capture the specific quality-of-life dynamics of Chinese patients with ES-SCLC during actual treatment, and the heterogeneity across different studies may lead to potential biases. Second, regarding cost accounting, this study adopted the perspective of the Chinese healthcare system and included only direct medical costs, omitting indirect costs associated with productivity loss or the caregiver burden on patient families. In addition, adverse event costs were modeled primarily during the initial treatment cycle, which may not fully capture the long-term management costs of certain persistent or recurrent immune-related adverse events.

Finally, several methodological limitations related to the machine learning models should be acknowledged. First, the DeepSurv and RSF models in this study incorporated only treatment assignment as a covariate and were essentially designed for population-level survival extrapolation rather than individualized risk prediction. Therefore, the present study could not further explore patient heterogeneity, individualized treatment benefit, or precision prediction, which are among the major potential advantages of machine learning approaches in healthcare applications. Second, although machine learning models may offer advantages in fitting complex nonlinear relationships, there remains a potential risk of overfitting during long-term survival extrapolation. In addition, compared with conventional parametric survival models, DeepSurv and RSF lack explicit parametric functional forms, making the clinical interpretability of their long-term hazard patterns relatively limited. Third, although the results across different models remained generally consistent, the current analysis was based solely on internal validation and lacked external validation using independent patient cohorts. Therefore, the true accuracy of the long-term survival extrapolations still requires confirmation through future real-world studies with extended follow-up durations. Fourth, although the C-index values were relatively low, calibration performance is generally considered more important than individual risk discrimination in health economic evaluations involving survival extrapolation. In the present study, all models maintained relatively low IBS values, indicating good agreement between the model-generated survival curves and the original clinical trial data, thereby providing a relatively stable basis for long-term survival extrapolation.

## 5 Conclusions

In summary, first-line tislelizumab plus chemotherapy for ES-SCLC aligns with current pharmacoeconomic evaluation standards in China. Following multidimensional validation with machine learning models, the robust cost-effectiveness of this regimen has been further substantiated. We recommend its active application in clinical practice based on individual patient circumstances, alongside dynamic adjustments to healthcare policies in conjunction with national medical insurance negotiations.

## Data Availability

The original contributions presented in the study are included in the article/Supplementary material, further inquiries can be directed to the corresponding author.
