# Supplementary material for: Pharmacoeconomic evaluation of first-line tislelizumab for extensive-stage small cell lung cancer using a comparative validation of traditional survival and machine learning models
Source: Front Public Health. 2026 Jun 17;14:1841510. doi: 10.3389/fpubh.2026.1841510 (PMC13318599; doi:10.3389/fpubh.2026.1841510)
Supplement: Supplementary file 1 [file Table_1.docx]

| **Table S1. Summary of hyperparameter configurations used in DeepSurv and RSF models** | | | |
| --- | --- | --- | --- |
|  | Category | Specification | Value |
| DeepSurv | Architecture | Total Hidden Layers | 3 |
|  |  | Nodes per Layer | 32 → 64 → 32 |
|  |  | Activation Function | ReLU (Rectified Linear Unit) |
|  | Optimization | Optimizer | Adam (Adaptive Moment Estimation) |
|  |  | Learning Rate | 0.001 |
|  |  | Batch Size | 32 |
|  |  | Training Epochs | 100 |
|  | Regularization | Batch Normalization | Applied after 1st and 2nd hidden layers |
|  |  | Dropout Rate | 0.1 (applied at the 2nd hidden layer) |
|  |  | Loss Function | Partial Likelihood of Cox Model |
| RSF | Forest Size | Number of Trees (n_estimators) | 100 |
|  | Splitting Rule | Minimum Samples to Split | 10 |
|  |  | Minimum Samples at Leaf | 5 |
|  | Tree Depth | Maximum Depth | Unconstrained (expanded until leaf constraints) |
|  | Parallelism | CPU Cores Used (n_jobs) | All available (-1) |
|  | Reproducibility | Random State Seed | 42 |

| **Table S2. AIC and BIC values for Survival curve fitting** | | | |  |  |
| --- | --- | --- | --- | --- | --- |
|  |  | Loglogistic | Lognormal | Gompertz | Weibull |
| AIC | PFS of placebo group | 1995.034 | 2100.314 | 2336.762 | 2439.234 |
|  | PFS of tislelizumab group | 2578.537 | 2618.049 | 2625.589 | 2782.982 |
|  | OS of placebo group | 3115.87 | 3123.47 | 3170.904 | 3227.144 |
|  | OS of tislelizumab group | 2929.523 | 2941.563 | 2975.143 | 2989.48 |
|  |  |  |  |  |  |
| BIC | PFS of placebo group | 2003.279 | 2108.559 | 2345.007 | 2447.479 |
|  | PFS of tislelizumab group | 2586.787 | 2626.298 | 2633.838 | 2791.231 |
|  | OS of placebo group | 3124.115 | 3131.715 | 3179.149 | 3235.389 |
|  | OS of tislelizumab group | 2937.772 | 2949.812 | 2983.392 | 2997.729 |

| \| **Table S3. Model performance evaluation metrics for OS and PFS across three survival modeling frameworks.** \| \| \| \| \| --- \| --- \| --- \| --- \| \| Model \| Endpoint \| C-index \| IBS \| \| DeepSurv \| OS \| 0.5250 (0.5010-0.5576) \| 0.1621 \| \| PFS \| 0.5224 (0.5064-0.5619) \| 0.0895 \| \|  \|  \|  \|  \| \| RSF \| OS \| 0.5255 (0.4985-0.5527) \| 0.1621 \| \| PFS \| 0.5113 (0.4946-0.5541) \| 0.0894 \| \|  \|  \|  \|  \| \| log-logistc \| OS \| 0.5294 (0.5101-0.5487) \| 0.1626 \| \| PFS \| 0.5444 (0.5253-0.5635) \| 0.0935 \|   IBS, Integrated Brier Score；RSF, Random Survival Forest |
| --- | --- | --- | --- | --- | --- | --- | --- | --- | --- | --- | --- | --- | --- | --- | --- | --- | --- | --- | --- | --- | --- | --- | --- | --- | --- | --- | --- | --- | --- | --- | --- | --- | --- | --- | --- | --- | --- |

| **Table S4. Comparison of cost-effectiveness results between 10-year and 5-year time horizons** | | | | | | | | | | |
| --- | --- | --- | --- | --- | --- | --- | --- | --- | --- | --- |
|  | Model | Group | Total cost  (CNY) | Incremental cost (CNY) | △%Incremental cost  (CNY) | QALYs | Incremental QALY | △%Incremental QALY | ICER  (CNY/QALY） | △%ICER  (CNY/QALY) |
| 10years | Log-logistic | Placebo group | 153,229.20 | 101,506.90 |  | 0.7073 | 0.4044 |  | 251,030.50 |  |
|  |  | Tislelizumab group | 254,736.10 |  |  | 1.1117 |  |  |  |  |
|  |  |  |  |  |  |  |  |  |  |  |
|  | DeepSurv | Placebo group | 130,453.03 | 69,000.06 |  | 0.5298 | 0.2636 |  | 261,718.45 |  |
|  |  | Tislelizumab group | 199,453.09 |  |  | 0.7934 |  |  |  |  |
|  |  |  |  |  |  |  |  |  |  |  |
|  | RSF | Placebo group | 114,264.31 | 115,092.34 |  | 0.4566 | 0.4635 |  | 248,299.41 |  |
|  |  | Tislelizumab group | 229,356.65 |  |  | 0.9201 |  |  |  |  |
|  |  |  |  |  |  |  |  |  |  |  |
| 5years | Log-logistic | Placebo group | 149,504.90 | 94,752.30 | 6.6543 | 0.6812 | 0.3648 | 9.7923 | 259,712.17 | 3.4584 |
|  |  | Tislelizumab group | 244,257.20 |  |  | 1.046 |  |  |  |  |
|  |  |  |  |  |  |  |  |  |  |  |
|  | DeepSurv | Placebo group | 120,503.43 | 62,876.76 | 8.8743 | 0.4779 | 0.2355 | 10.6601 | 266,922.75 | 1.9885 |
|  |  | Tislelizumab group | 183,380.19 |  |  | 0.7134 |  |  |  |  |
|  |  |  |  |  |  |  |  |  |  |  |
|  | RSF | Placebo group | 113,035.38 | 104,865.91 | 8.8854 | 0.4508 | 0.4137 | 10.7443 | 253,469.38 | 2.0822 |
|  |  | Tislelizumab group | 217,901.29 |  |  | 0.8645 |  |  |  |  |

| **Table S5. One-way sensitivity analysis for the three models** | | | | | | |
| --- | --- | --- | --- | --- | --- | --- |
|  | Parameter | Base Value | Lower Limit | Upper Limit | ICER  (Lower Limit) | ICER  (Upper Limit) |
| Log-logistic | Utility PFS | 0.673 | 0.538 | 0.808 | 287865.91 | 222552.55 |
|  | Cost of tislelizumab | 2755 | 2204 | 3306 | 231232.91 | 270828.02 |
|  | Utility PD | 0.473 | 0.378 | 0.568 | 267919.95 | 236144.11 |
|  | Risk of thrombocytopenia in tislelizumab group | 0.19 | 0.152 | 0.228 | 236108.88 | 266016.7 |
|  | Cost of tumor imaging per cycle | 3609.84 | 2887.87 | 4331.81 | 239168.87 | 262892.06 |
|  | Risk of thrombocytopenia in placebo group | 0.25 | 0.2 | 0.3 | 262067.3 | 240056.23 |
|  | Risk of leukopenia in tislelizumab group | 0.24 | 0.192 | 0.288 | 242432.8 | 259861.92 |
|  | Discount rate | 0.05 | 0 | 0.08 | 240423.8 | 257342.03 |
|  | Risk of neutropenia in placebo group | 0.21 | 0.168 | 0.252 | 258923.37 | 243458.82 |
|  | Cost of best supportive treatment per cycle | 3155.5 | 2524.4 | 3786.59 | 243771.61 | 258289.2 |
|  | Risk of leukopenia in placebo group | 0.27 | 0.216 | 0.324 | 258190.69 | 244083.13 |
|  | Risk of neutropenia in tislelizumab group | 0.14 | 0.112 | 0.168 | 244555.39 | 257687.41 |
|  | Risk of anemia in tislelizumab group | 0.16 | 0.128 | 0.192 | 245545.68 | 256578.99 |
|  | Cost of thrombocytopenia | 10555 | 8444 | 12666 | 246910.73 | 255150.2 |
|  | Risk of anemia in placebo group | 0.17 | 0.136 | 0.204 | 254940.16 | 247168.47 |
|  | Proportion of subsequent anticancer therapy in tislelizumab group | 0.55 | 0.44 | 0.66 | 247627.81 | 254433.12 |
|  | Proportion of subsequent anticancer therapy in placebo group | 0.67 | 0.536 | 0.8 | 254325.3 | 247833.98 |
|  | Cost of carboplatin | 160.62 | 125.21 | 750.5 | 250739.58 | 255876.18 |
|  | Cost of leukopenia | 3099.6 | 2479.68 | 3719.52 | 248948.74 | 253112.19 |
|  | Cost of topotecan | 551.64 | 551.58 | 4310.75 | 251030.41 | 254704.13 |
|  |  |  |  |  |  |  |
| DeepSurv | Utility PFS | 0.673 | 0.538 | 0.808 | 298231.64 | 233170.81 |
|  | Cost of tislelizumab | 2755 | 2204 | 3306 | 236371.56 | 287065.34 |
|  | Risk of thrombocytopenia in tislelizumab group | 0.19 | 0.152 | 0.228 | 242463.87 | 281101.12 |
|  | Utility PD | 0.473 | 0.378 | 0.568 | 279435.99 | 246113.7 |
|  | Risk of thrombocytopenia in placebo group | 0.25 | 0.2 | 0.3 | 278916.35 | 244669.93 |
|  | Risk of neutropenia in placebo group | 0.21 | 0.168 | 0.252 | 274364.19 | 249853.65 |
|  | Risk of leukopenia in tislelizumab group | 0.24 | 0.192 | 0.288 | 249736.39 | 274203.89 |
|  | Risk of leukopenia in placebo group | 0.27 | 0.216 | 0.324 | 273108.07 | 250844.13 |
|  | Cost of tumor imaging per cycle | 3609.84 | 2887.87 | 4331.81 | 250369.3 | 273067.61 |
|  | Risk of neutropenia in tislelizumab group | 0.14 | 0.112 | 0.168 | 252403.3 | 271437.91 |
|  | Risk of anemia in tislelizumab group | 0.16 | 0.128 | 0.192 | 254259.53 | 269310.73 |
|  | Cost of best supportive treatment per cycle | 3155.5 | 2524.4 | 3786.59 | 254417.53 | 269019.26 |
|  | Risk of anemia in placebo group | 0.17 | 0.136 | 0.204 | 267881.59 | 255670.27 |
|  | Cost of carboplatin | 160.62 | 125.21 | 750.5 | 261440.13 | 266354.82 |
|  | Cost of topotecan | 551.64 | 551.58 | 4310.75 | 261718.38 | 266170.92 |
|  | Discount rate | 0.05 | 0 | 0.08 | 257939.51 | 263910.94 |
|  | Proportion of subsequent anticancer therapy in placebo group | 0.67 | 0.536 | 0.8 | 264908.25 | 258623.87 |
|  | Proportion of subsequent anticancer therapy in tislelizumab group | 0.55 | 0.44 | 0.66 | 258397.97 | 265038.93 |
|  | Utility neutropenia | 0.2 | 0.16 | 0.24 | 264527.86 | 258968.09 |
|  | Cost of thrombocytopenia | 10555 | 8444 | 12666 | 259248.94 | 264187.96 |
|  |  |  |  |  |  |  |
| RSF | Utility PFS | 0.673 | 0.538 | 0.808 | 288097.89 | 218162.03 |
|  | Cost of tislelizumab | 2755 | 2204 | 3306 | 230988.07 | 265610.75 |
|  | Utility PD | 0.473 | 0.378 | 0.568 | 262495.42 | 235560.09 |
|  | Risk of thrombocytopenia in tislelizumab group | 0.19 | 0.152 | 0.228 | 235254.37 | 261393.75 |
|  | Cost of tumor imaging per cycle | 3609.84 | 2887.87 | 4331.81 | 235493.99 | 261104.83 |
|  | Cost of topotecan | 551.64 | 551.58 | 4310.75 | 248299.26 | 257965.95 |
|  | Risk of thrombocytopenia in placebo group | 0.25 | 0.2 | 0.3 | 256155.22 | 240482.49 |
|  | Risk of leukopenia in tislelizumab group | 0.24 | 0.192 | 0.288 | 240807.16 | 255969.09 |
|  | Discount rate | 0.05 | 0 | 0.08 | 241069.88 | 252743.03 |
|  | Risk of neutropenia in placebo group | 0.21 | 0.168 | 0.252 | 254678.6 | 242147.32 |
|  | Cost of best supportive treatment per cycle | 3155.5 | 2524.4 | 3786.59 | 242072.09 | 254526.63 |
|  | Risk of neutropenia in tislelizumab group | 0.14 | 0.112 | 0.168 | 242667.25 | 254069.33 |
|  | Risk of leukopenia in placebo group | 0.27 | 0.216 | 0.324 | 253932.23 | 242812.97 |
|  | Cost of thrombocytopenia | 10555 | 8444 | 12666 | 242918.24 | 253680.59 |
|  | Cost of carboplatin | 160.62 | 125.21 | 750.5 | 247985.38 | 253530.7 |
|  | Risk of anemia in tislelizumab group | 0.16 | 0.128 | 0.192 | 243516.55 | 253130.72 |
|  | Risk of anemia in placebo group | 0.17 | 0.136 | 0.204 | 251290.08 | 245340.6 |
|  | Cost of leukopenia | 3099.6 | 2479.68 | 3719.52 | 245914.96 | 250683.87 |
|  | Proportion of subsequent anticancer therapy in tislelizumab group | 0.55 | 0.44 | 0.66 | 246256.68 | 250342.15 |
|  | Cost of anemia | 3536.6 | 2829.28 | 4243.92 | 246388.89 | 250209.93 |
